# Supplementary material for: Palladium(II)-Catalyzed Efficient Synthesis of Wedelolactone and Evaluation as Potential Tyrosinase Inhibitor
Source: Molecules. 2019 Nov 15;24(22):4130. doi: 10.3390/molecules24224130 (PMC6891477; doi:10.3390/molecules24224130)
Supplement: Supplementary file 1 [file molecules-24-04130-s001.pdf]

Palladium (II)-catalyzed efficient synthesis of Wedelolactone and evaluation as potential tyrosinase inhibitor

Huidan Huang[a], Jianqiu Chen [a,*], Jie Ren[b], Chaofeng Zhang[b,*], and Fei Ji[a,*]

[a]College of Engineering, China Pharmaceutical University, #639 Longmian Avenue, Jiangning District, Nanjing, 211198, China, email: jifei672453142@126.com
[b]State Key Laboratory of Natural Medicines, Research Department of Pharmacognosy, China Pharmaceutical University, 639 Longmian Road, Nanjing, 211198, China.

Table of contents

Optimization of boronation/Suzuki-Miyaura reactions

^{1}H NMR and ^{13}C NMR spectra of the products

Optimization of boronation/Suzuki-Miyaura reactions[a]

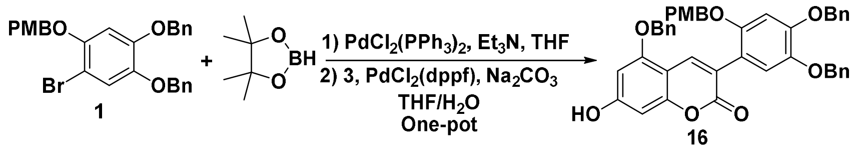

Entry	n_1:n_3[b]	$Pd(PPh_3)_2Cl_2$(mol%)	Pd(dppf)Cl_2(mol%)	THF(mL/mmol)	Yield[c] of **16** (%)
1	1.1	10	10	20	45
2	1.3	10	10	20	54
3	1.5	10	10	20	75
4	1.7	10	10	20	71
5	1.5	5	10	20	73
6	1.5	5	5	20	70
7	1.5	3	5	20	61
8[d]	1.5	5	5	20	42
9	1.5	5	5	10	72
10	1.5	5	5	5	62

[a]Reaction conditions: **1** (1.0 mmol), pinacoborane (7.5 mmol), $Pd(PPh_3)_2Cl_2$, TEA (8.6 mmol), THF, under Ar atmosphere, stirred at 80 ℃ overnight; **3**, Pd(dppf)Cl_2, Na_2CO_3 (7 mmol), H_2O (2.5 mL/mmol), under Ar atmosphere, stirred at 90 ℃ for another 12 h.

[b]The ratio of bromide product **1** and bromocoumarin **3**.

[c]Isolated yield.

[d]Under air atmosphere.

^{1}H NMR and ^{13}C NMR spectra of the products

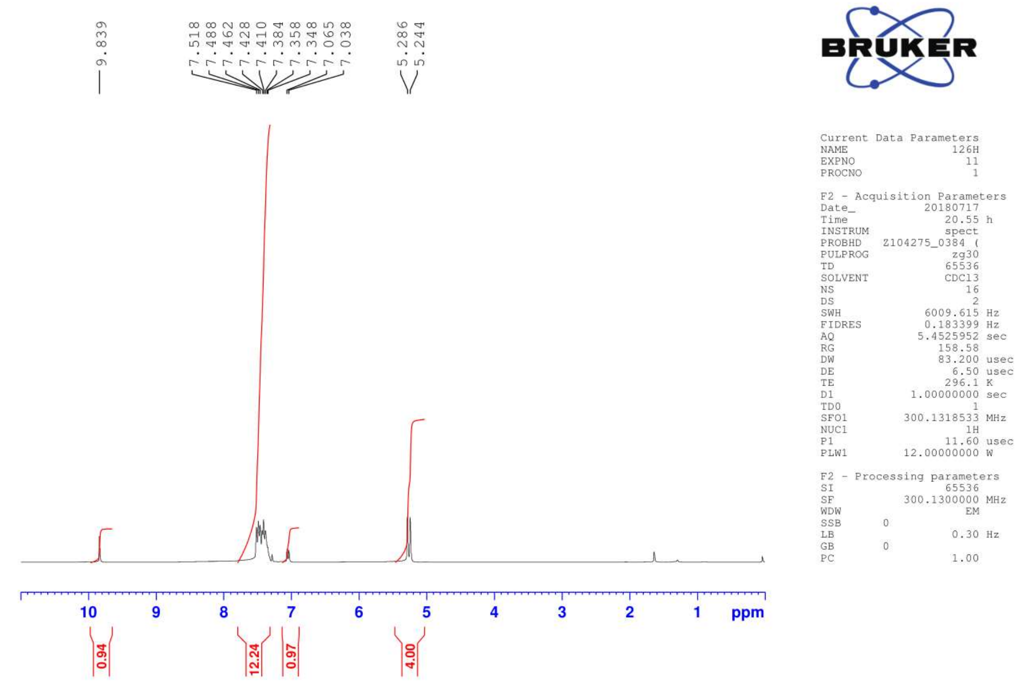

^{1}H NMR of **7**

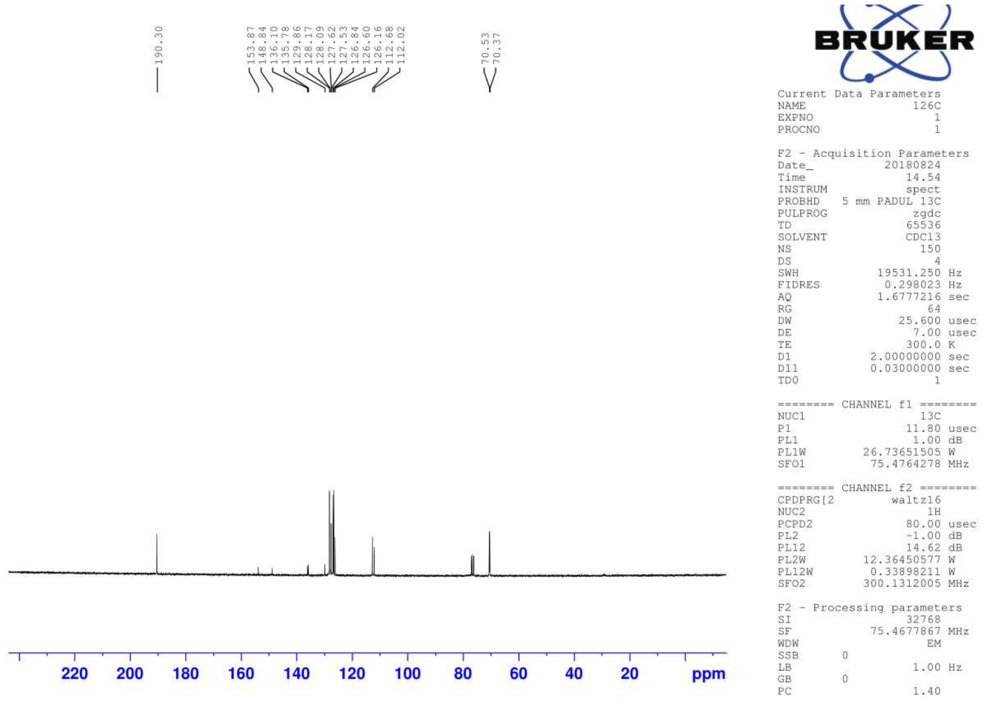

^{13}C NMR of **7**

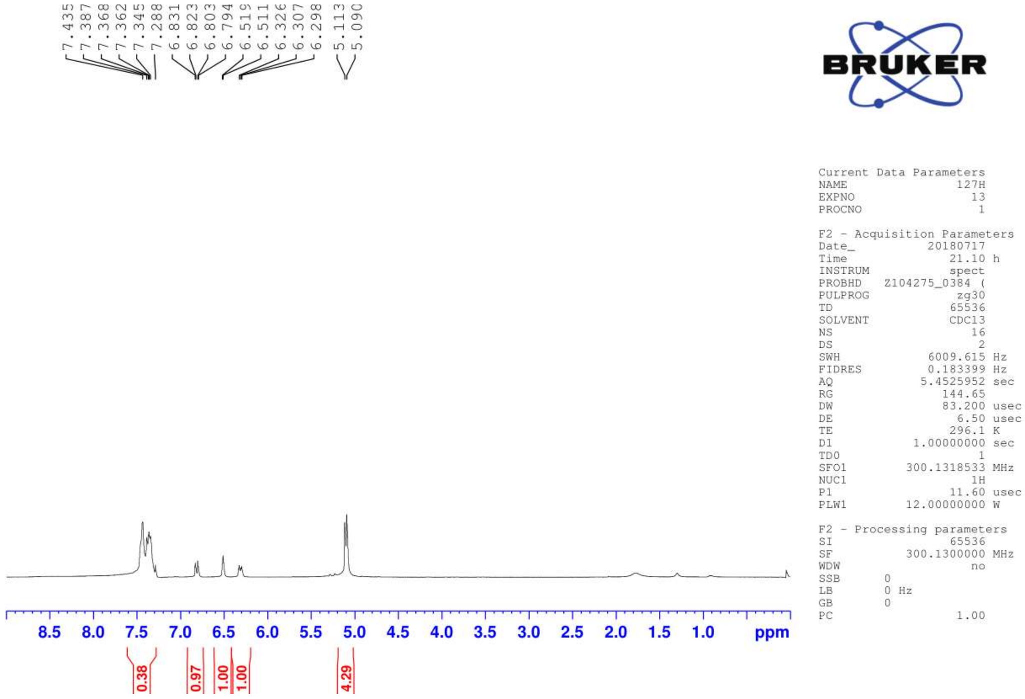

^{1}H NMR of **8**

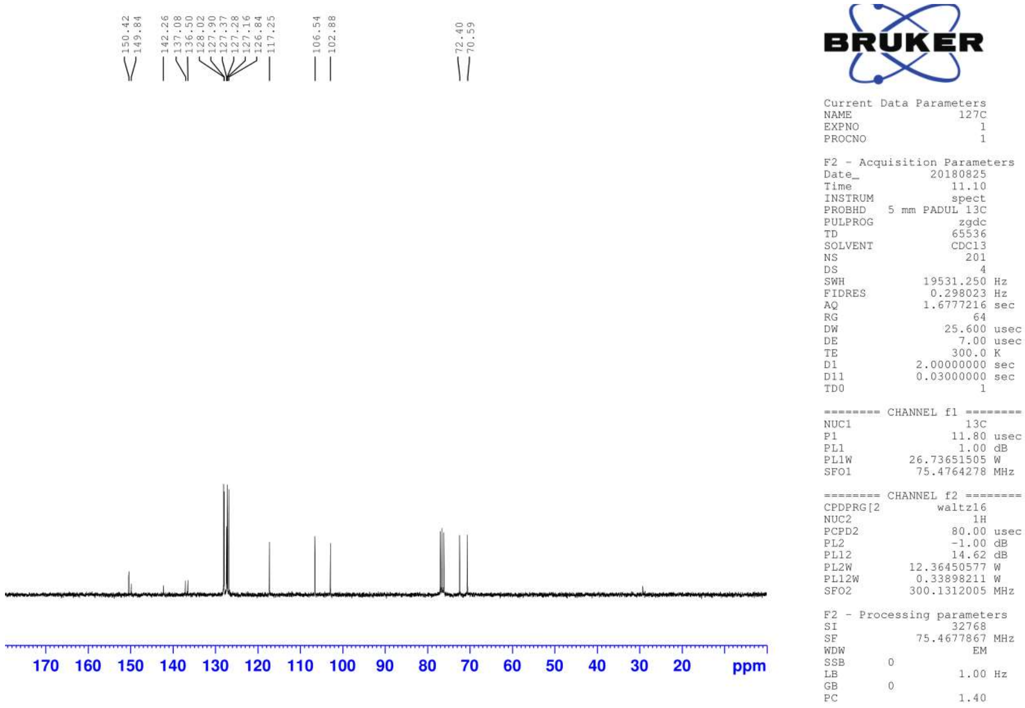

^{13}C NMR of **8**

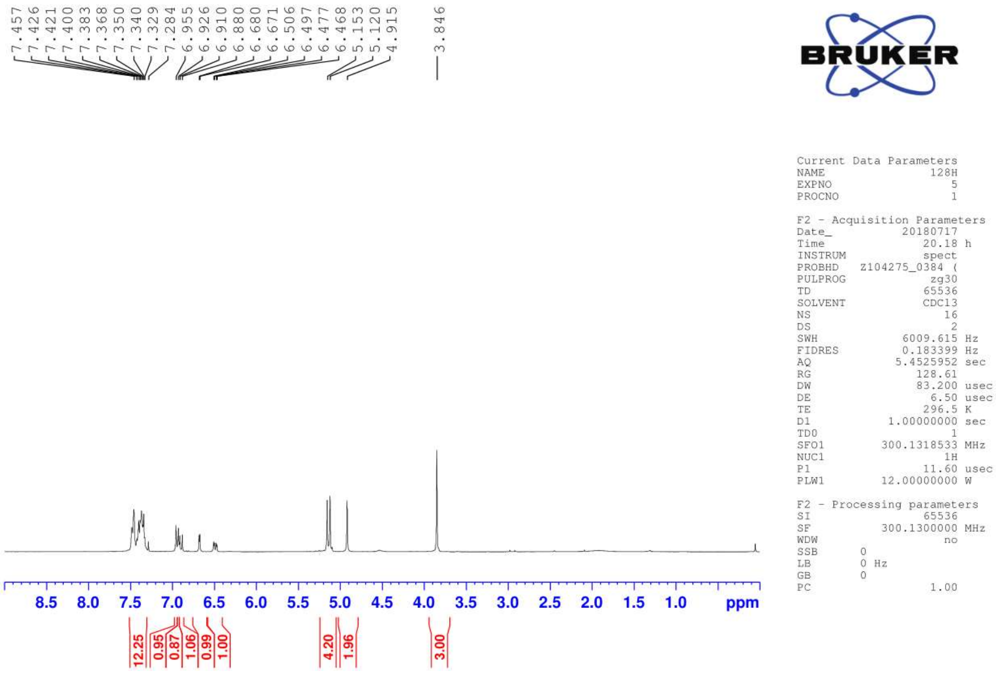

^{1}H NMR of **9**

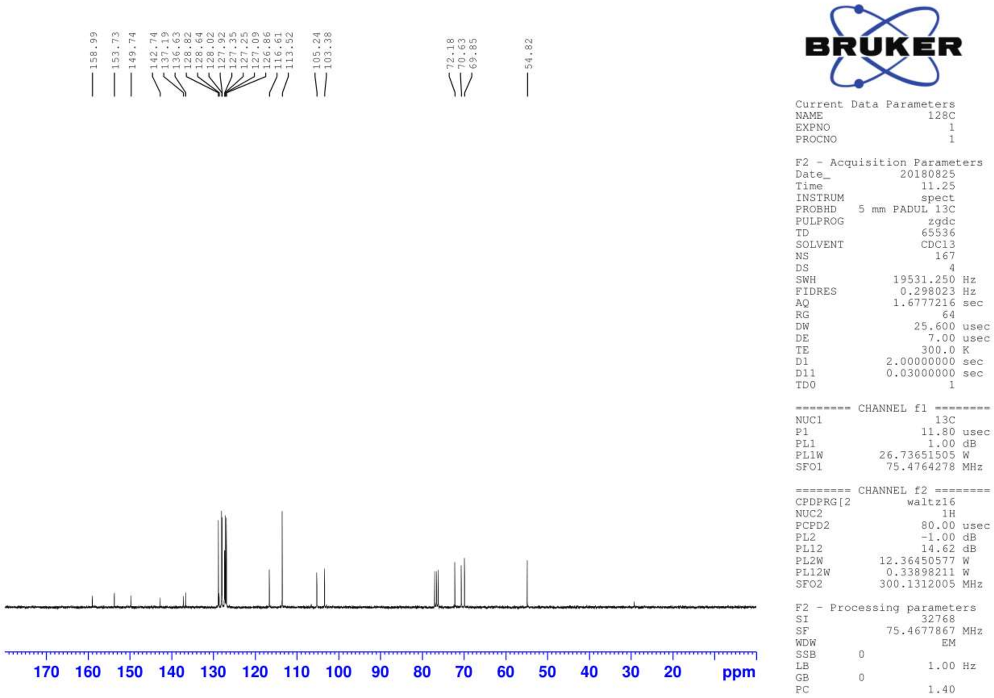

^{13}C NMR of **9**

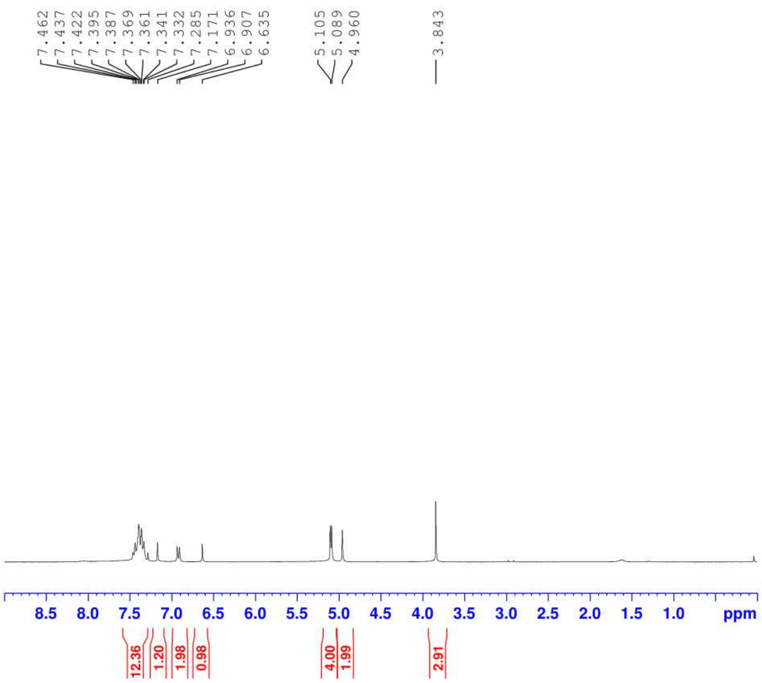


```
Current Data Parameters
NAME            129H
EXPNO             10
PROCNO             1

F2 - Acquisition Parameters
Date_       20180717
Time           20.48 h
INSTRUM        spect
PROBHD   Z104275_0384 (
PULPROG         zg30
TD             65536
SOLVENT        CDCl3
NS                16
DS                 2
SWH         6009.615 Hz
FIDRES      0.183399 Hz
AQ         5.4525952 sec
RG            158.58
DW            83.200 usec
DE              6.50 usec
TE             296.3 K
D1        1.00000000 sec
TD0                1
SFO1     300.1318533 MHz
NUC1              1H
P1             11.60 usec
PLW1     12.00000000 W

F2 - Processing parameters
SI             65536
SF       300.1300000 MHz
WDW               no
SSB      0
LB       0 Hz
GB       0
PC              1.00
```

^{1}H NMR of **1**

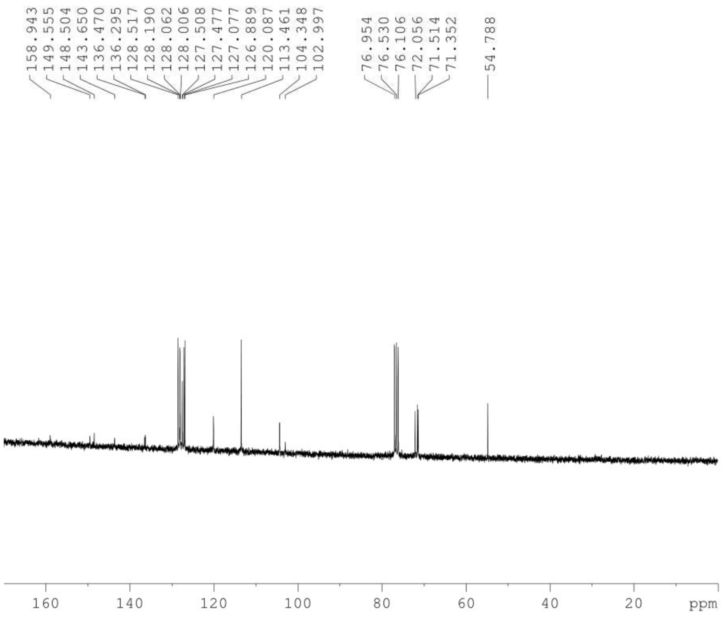


```
Current Data Parameters
NAME            129C
EXPNO              1
PROCNO             1

F2 - Acquisition Parameters
Date_       20180822
Time           14.17
INSTRUM        spect
PROBHD   5 mm PADUL 13C
PULPROG         zgdc
TD             65536
SOLVENT        CDCl3
NS               256
DS                 4
SWH        19531.250 Hz
FIDRES      0.298023 Hz
AQ         1.6777216 sec
RG                64
DW            25.600 usec
DE              7.00 usec
TE             300.0 K
D1        2.00000000 sec
D11       0.03000000 sec
TD0                1

======== CHANNEL f1 ========
NUC1             13C
P1             11.80 usec
PL1             1.00 dB
PL1W     26.73651505 W
SFO1      75.4764278 MHz

======== CHANNEL f2 ========
CPDPRG[2     waltz16
NUC2              1H
PCPD2          80.00 usec
PL2            -1.00 dB
PL12           14.62 dB
PL2W     12.36450577 W
PL12W     0.33898211 W
SFO2     300.1312005 MHz

F2 - Processing parameters
SI             32768
SF        75.4677867 MHz
WDW               EM
SSB      0
LB              1.00 Hz
GB       0
PC              1.40
```

^{13}C NMR of **1**

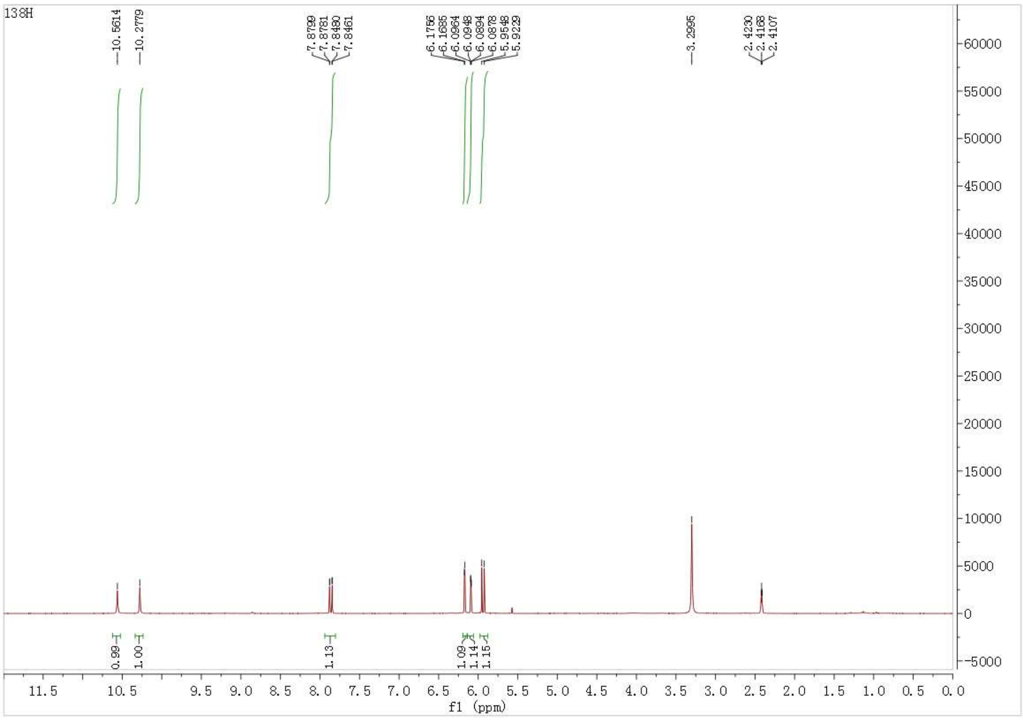

^{1}H NMR of **11**

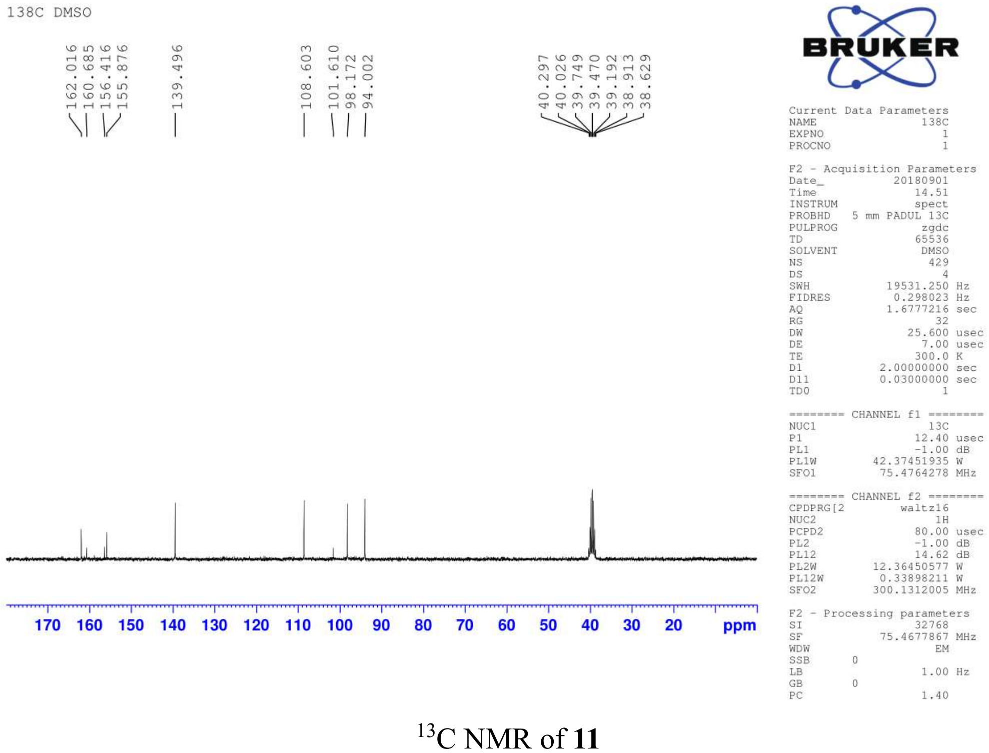

^{13}C NMR of **11**

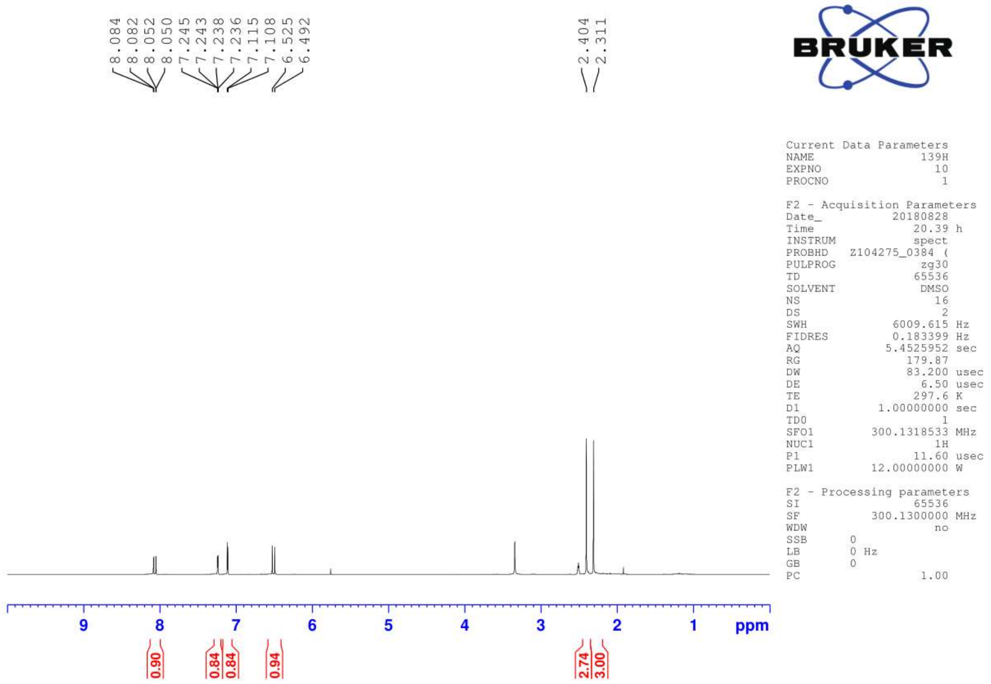

^{1}H NMR of **12**

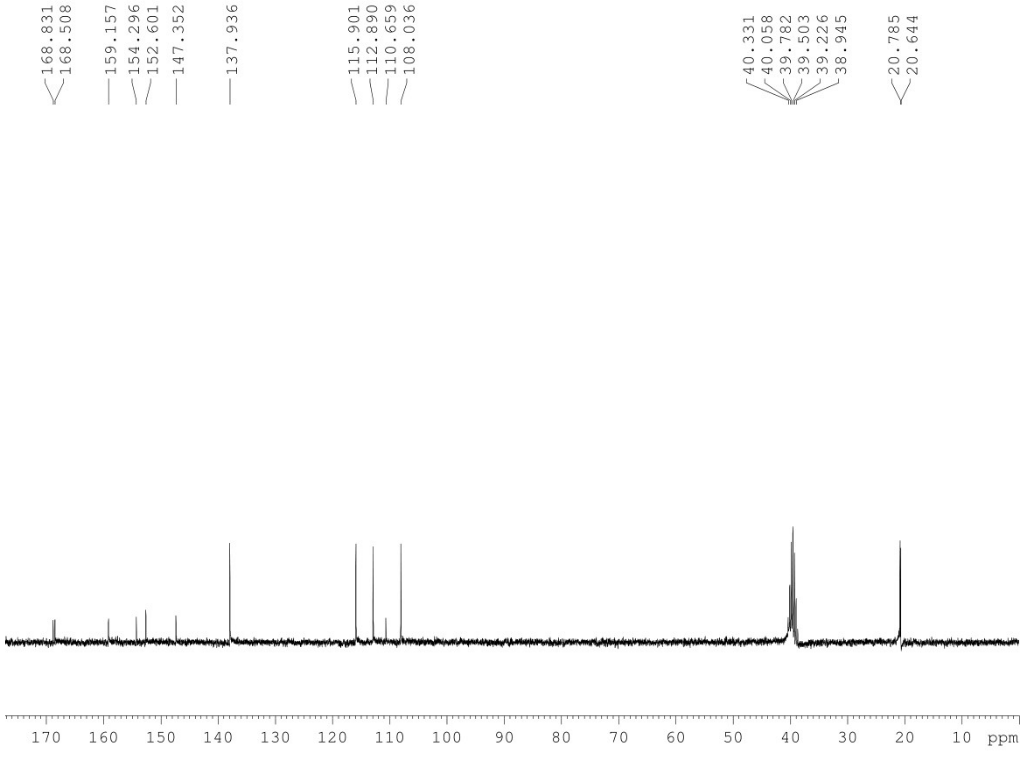

^{13}C NMR of **12**

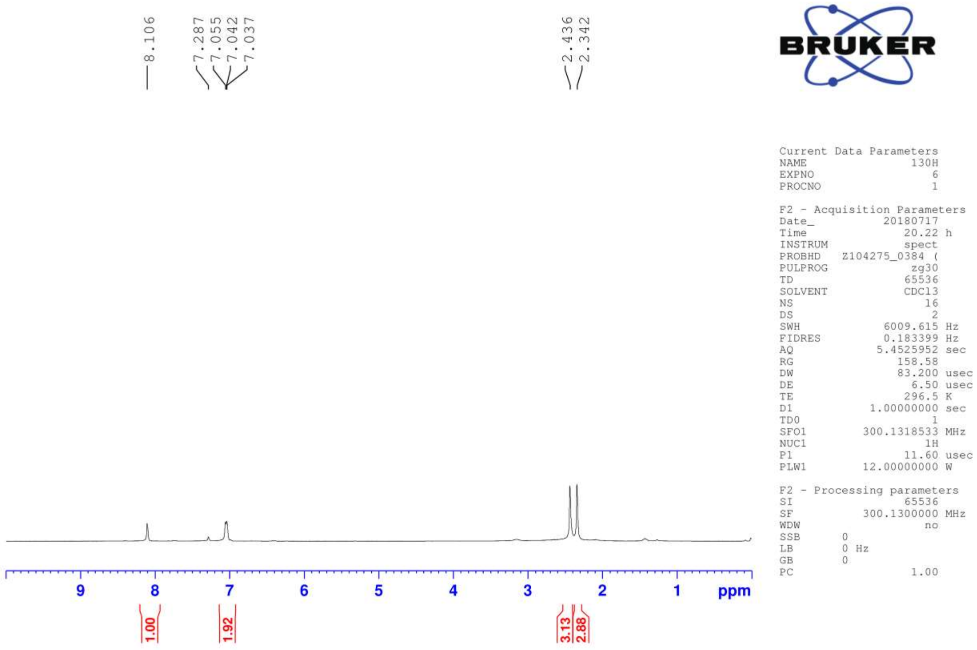

^{1}H NMR of **13**

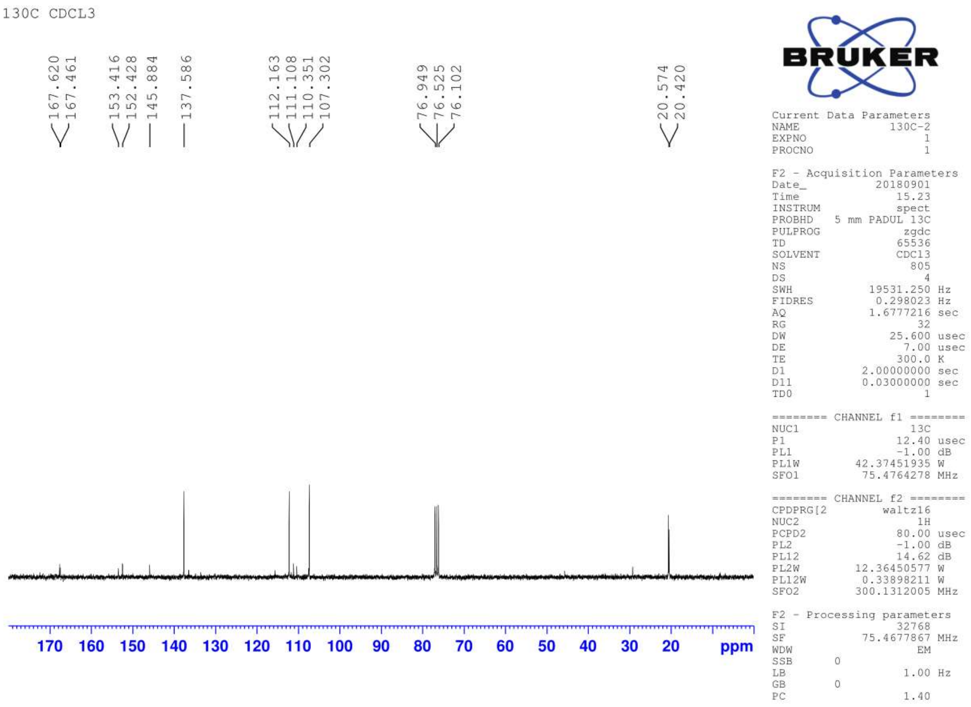

^{13}C NMR of **13**

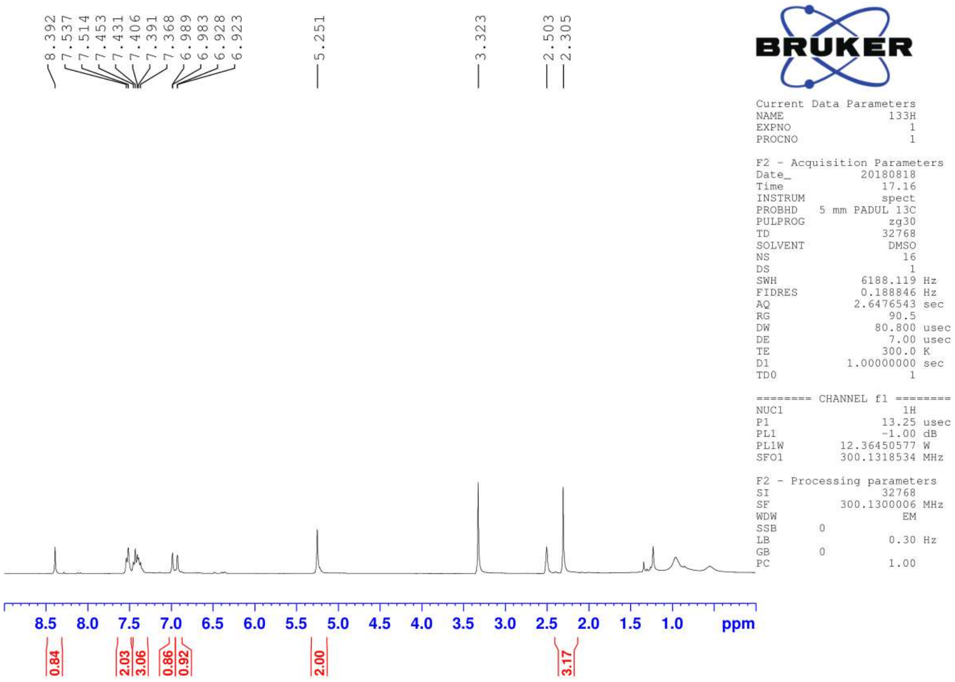

^{1}H NMR of **3**

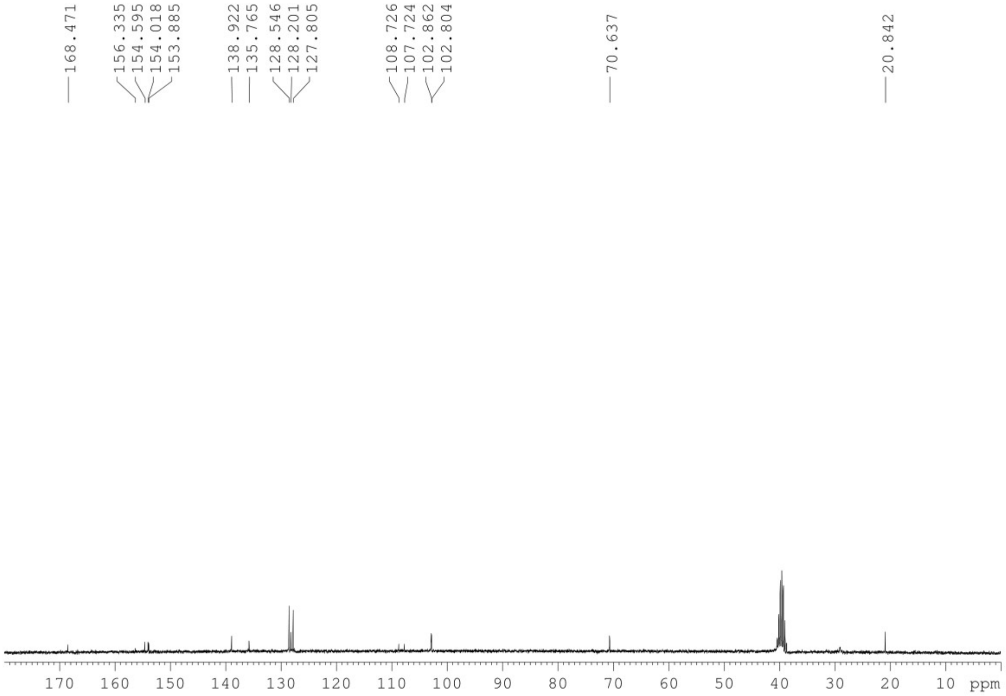

^{13}C NMR of **3**

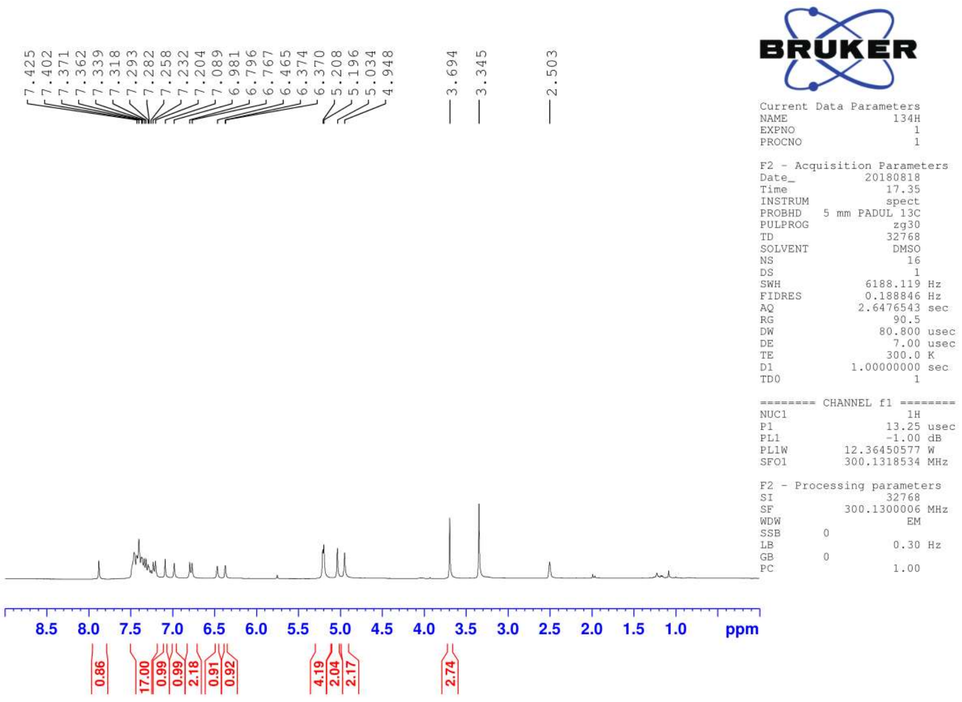

^{1}H NMR of **16**

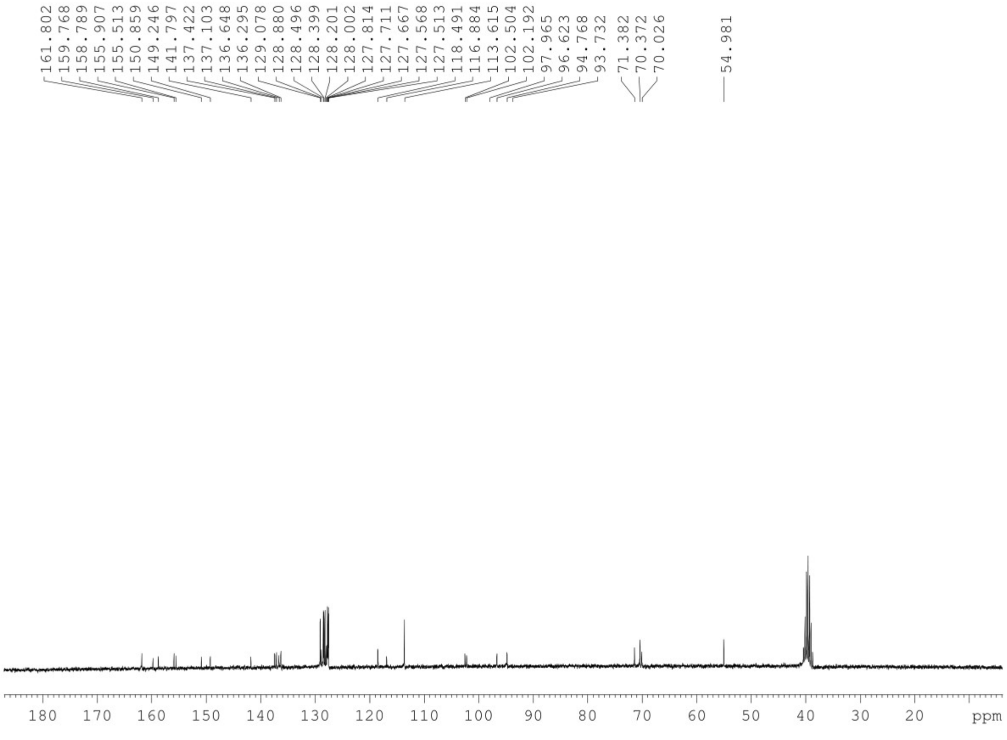

^{13}C NMR of **16**

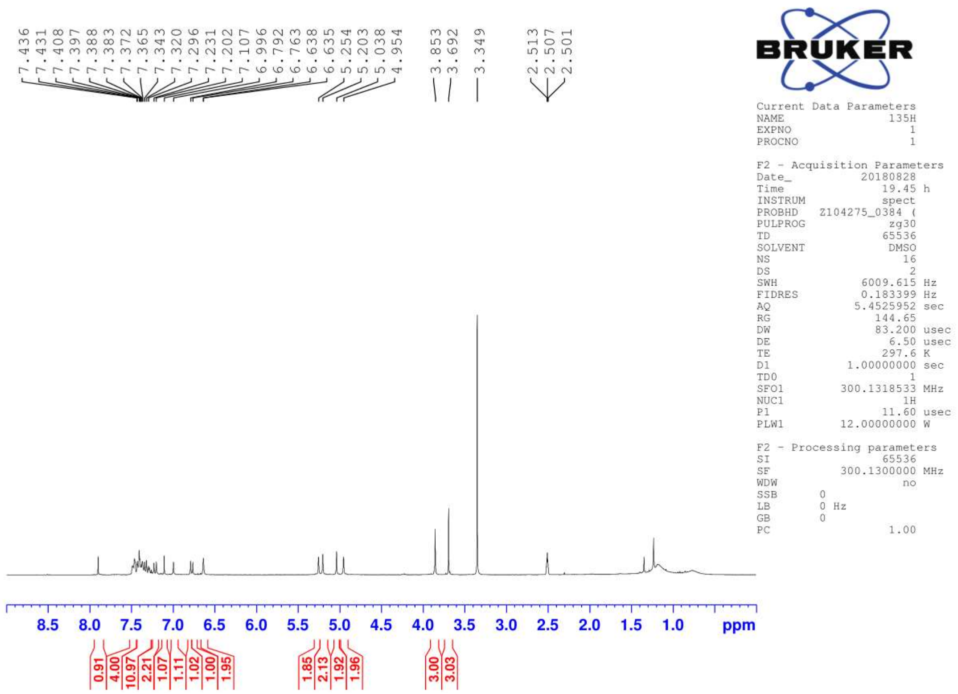

^{1}H NMR of **4**

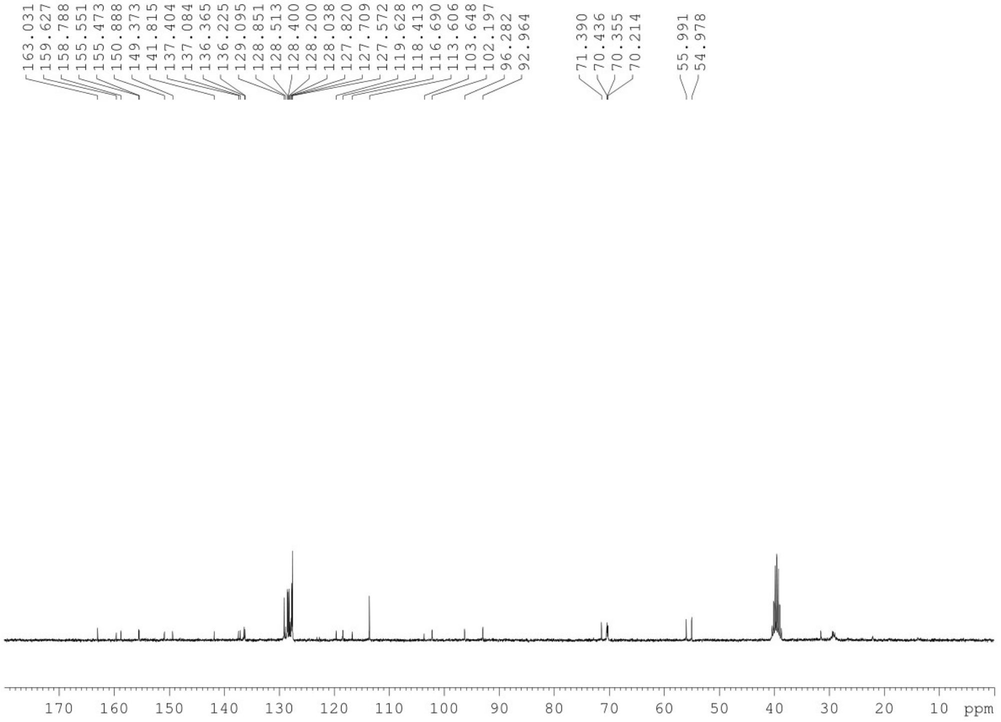

^{13}C NMR of **4**

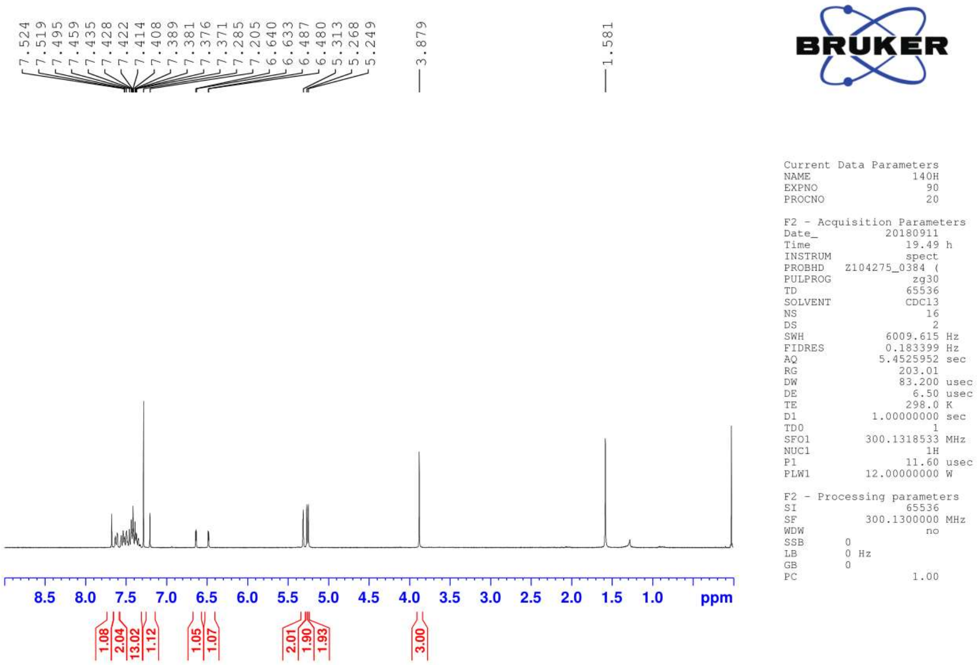

^{1}H NMR of **17**

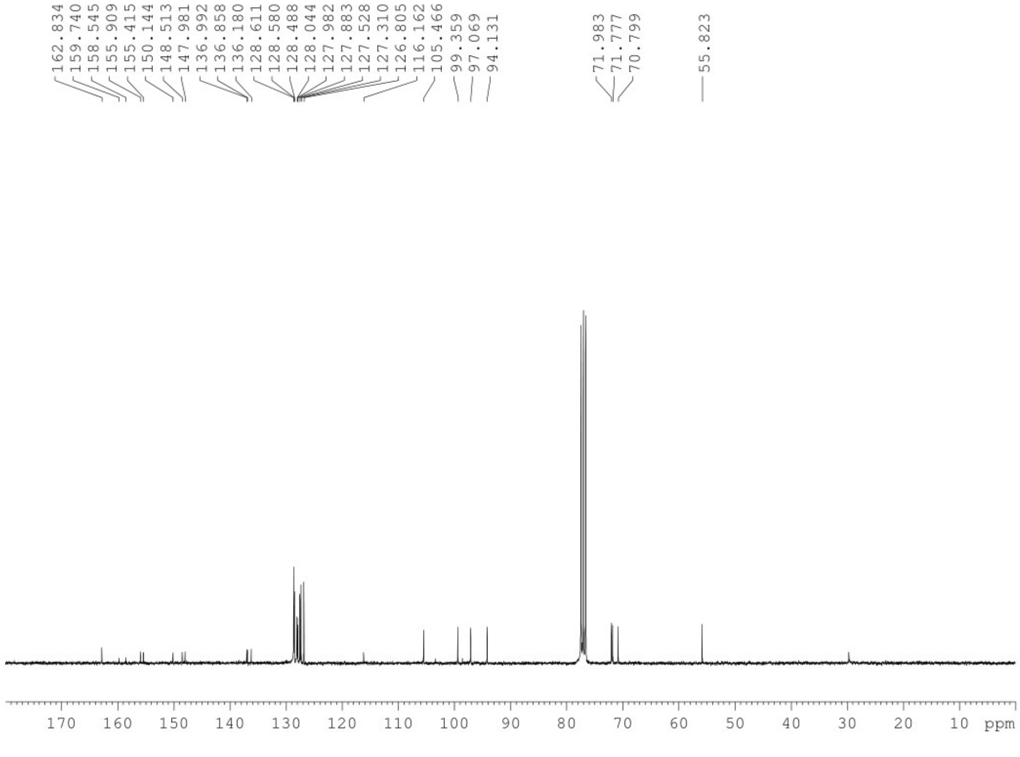

^{13}C NMR of **17**

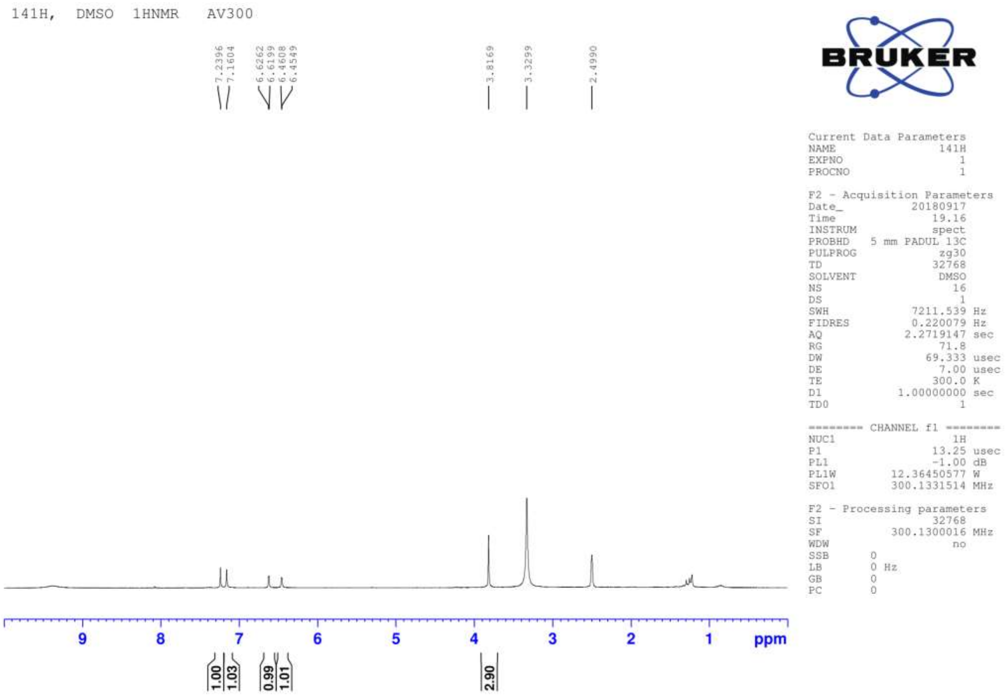

^{1}H NMR of **5**

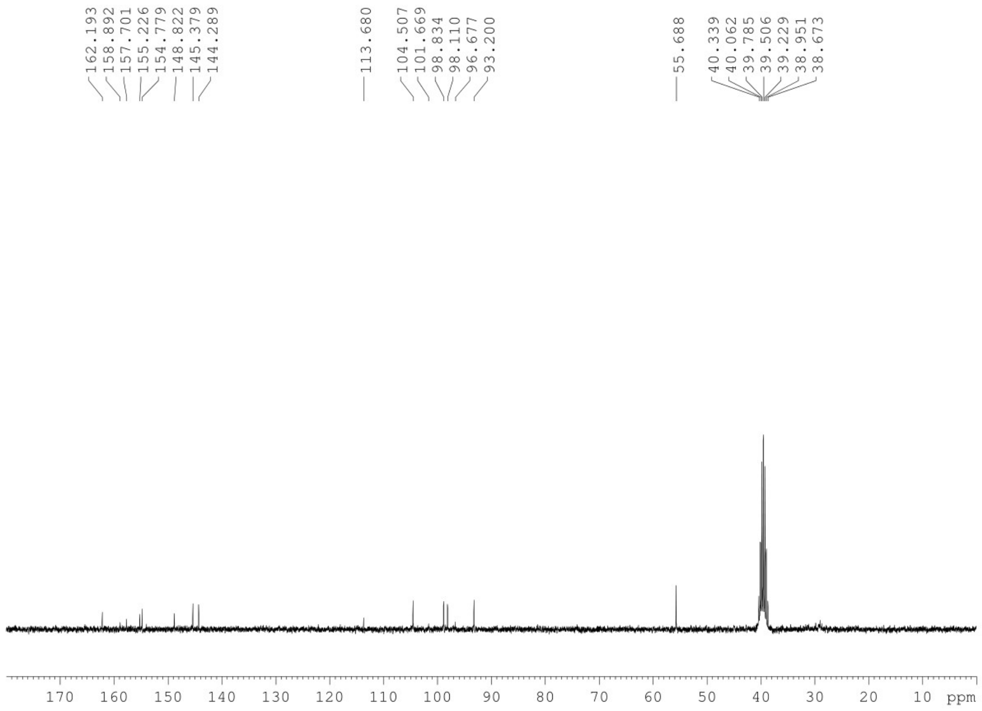

^{13}C NMR of **5**
